# Advances in veterinary drug and vaccine delivery: from conventional delivery methods to innovative transdermal approaches

**DOI:** 10.1007/s13346-026-02105-w

**Published:** 2026-04-20

**Authors:** Katherine A. Miranda-Muñoz, TsungCheng Tsai, Jeremy G. Powell, Jorge Almodovar

**Affiliations:** 1 Department of Biomedical Engineering, College of Engineering, University of Arkansas, Fayetteville, AR 72701 USA; 2 Department of Animal Sciences, Food and Life Sciences Building, University of Arkansas, B110 Agriculture, Fayetteville, AR 72701 USA; 3 Department of Chemical, Biochemical, and Environmental Engineering, University of Maryland Baltimore County, 1000 Hilltop Circle, Baltimore, MD 21250 USA

**Keywords:** Microneedle patches, Veterinary transdermal drug delivery, Biodegradable polymers, Pain management in animals, Sustained-release drug systems

## Abstract

Veterinary drug delivery must balance efficacy, ease of administration, and animal welfare. Traditional methods, such as oral and injectable routes, pose challenges due to species-specific variations, stress during administration, and the need for repeated dosing. Emerging transdermal systems, particularly microneedle patches, offer a minimally invasive alternative that enhances drug absorption while reducing handling stress toward animals and improving compliance. Microneedle technology enables controlled and sustained drug release, making it particularly effective for pain management, vaccine delivery, and hormone therapies. By facilitating the transdermal administration of analgesics, microneedles deliver long-lasting pain relief with fewer interventions, improving the treatment of acute and chronic conditions in veterinary medicine. The use of biodegradable materials further enhances safety, eliminates the need for patch removal, and minimizes environmental impact. However, challenges such as species-specific skin permeability, formulation stability, and regulatory approval must be addressed for widespread adoption. This review examines the evolution of veterinary drug delivery, emphasizing microneedle-based technologies and their potential to improve pain management and therapeutic outcomes. By overcoming existing limitations, these systems could transform treatment approaches, simplify administration, and enhance overall animal care. Continued research and development will be essential for optimizing these technologies and expanding their applications in both clinical and agricultural settings.

**Graphical abstract:**

**Figure:**
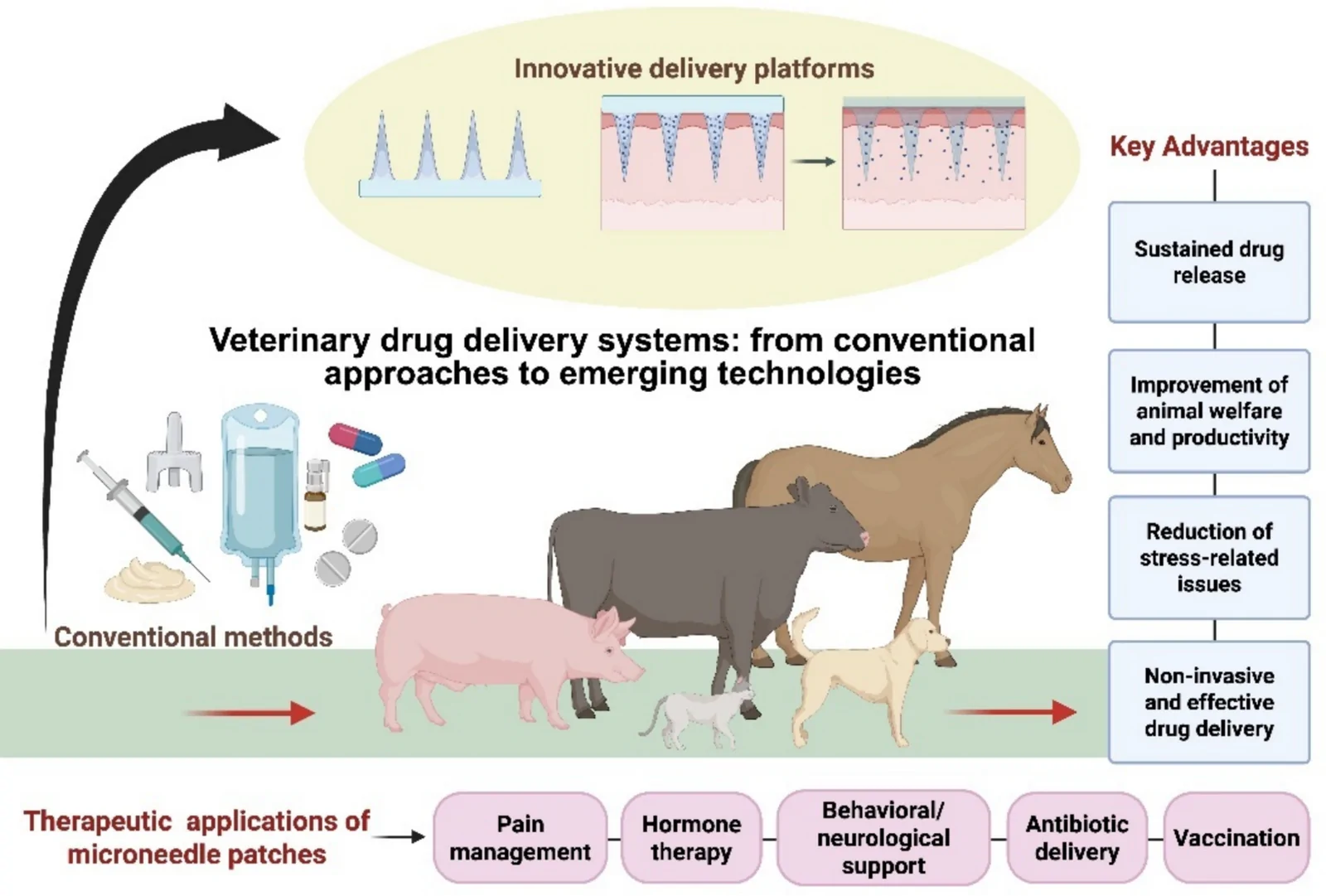

## Introduction

Effective drug delivery in veterinary medicine is essential for ensuring successful treatment outcomes while addressing the diverse physiological and behavioral characteristics of various animal species. Unlike human medicine, veterinary pharmacotherapy must accommodate a wide range of species, each with distinct anatomical, metabolic, and behavioral traits [1–3]. From companion animals like dogs and cats to livestock such as cattle, pigs, and horses, veterinarians must carefully select the most suitable delivery method to optimize therapeutic efficacy while minimizing stress and ensuring compliance. This decision is influenced by factors such as the medication's properties, the severity and urgency of the condition, species-specific absorption and metabolism, and regulatory guidelines that govern veterinary drug approval and usage [4–6].

Veterinary pharmaceuticals exist in multiple dosage forms, each designed to enhance drug efficacy while addressing the challenges of species-specific administration. While human patients can be instructed to follow precise dosing regimens, animals often require a minimum to moderate level of restraint during medication administration, which can be stressful for both animals and operators. [2, 6–8]. Traditional oral and injectable routes remain widely used, but advancements in veterinary drug delivery have introduced innovative technologies aimed at improving therapeutic outcomes and patient welfare [1, 6].

Among these innovations, transdermal delivery systems, such as microneedle patches, have gained attention for their ability to provide sustained, controlled drug release with minimal invasiveness [1]. Technologies including transdermal patches, hydrogel-based formulations, and microneedle arrays offer practical alternatives to frequent injections or oral administration, improving both compliance and treatment efficacy [9–12]. Hydrogels in particular can function either as standalone drug carriers, applied topically like a cream or as integral components of patch systems, serving as reservoirs or matrix structures for controlled drug release through the skin [13]. Microneedle patches have emerged as a promising approach for delivering vaccines, analgesics, and hormone therapies by creating microchannels in the skin, facilitating drug absorption with minimal discomfort [14]. The integration of biodegradable polymers and nanoparticle-based formulations further enhances these delivery systems, expanding their potential applications in veterinary medicine [15, 16].

Pain in animals is a major welfare and productivity concern, yet it is frequently underdiagnosed due to species-specific behavioral masking and communication barriers. Acute pain commonly arises from surgical interventions, trauma, and routine husbandry procedures, whereas chronic pain is associated with conditions such as osteoarthritis, inflammatory disorders, and neuropathies [7, 17–20]. Effective pain management improves animal welfare, postoperative recovery, and production metrics in livestock [18]. In this context, novel delivery platforms such as microneedle patches are particularly attractive for enabling sustained, low-stress analgesic delivery in veterinary patients.

Beyond the development of novel drug delivery systems, effective veterinary pharmacotherapy also depends on proper education and training for both veterinary professionals and animal caregivers [16, 21]. Incorrect administration can compromise treatment effectiveness, increase stress for the animal, and escalate veterinary costs due to therapeutic failures. Therefore, comprehensive training programs and clear administration guidelines are critical for ensuring optimal drug delivery in both clinical and at-home settings.

As veterinary medicine continues to evolve, the advancement of drug delivery technologies remains a key focus in enhancing treatment efficacy, reducing stress, and promoting overall animal health. This review provides a comprehensive analysis of veterinary drug delivery, examining classification, administration considerations, and emerging innovations that are shaping the future of veterinary pharmacotherapy.

## Drug and vaccine delivery in veterinary medicine: overview, classification, and administration considerations

The administration of drugs in veterinary practice is fundamental to ensuring effective treatment while addressing the diverse physiological and behavioral needs of various animal species [5]. Whether treating companion animals like dogs and cats or managing the health of livestock such as cattle, pigs, and horses, choosing the optimal drug delivery route is critical to ensuring therapeutic success [5, 22]. This decision depends on factors such as the type of medication, the species being treated, the severity of the condition, and the need for rapid or sustained therapeutic effects. Regulatory frameworks also influence the selection and approval of veterinary drugs to ensure safety and efficacy in different species [23, 24].

Veterinary medicine employs a range of pharmaceutical dosage forms, each tailored to the unique physiological and behavioral characteristics of different animals. These formulations are designed to optimize drug efficacy, safety, and compliance by accounting for species-specific factors such as digestive physiology, metabolic rates, and typical behavioral responses [5, 22, 25–27]. Additionally, ensuring that treatment is both effective and minimally stressful aligns with the principles of animal welfare, reducing discomfort while maximizing therapeutic benefits [28, 29]. The selection of an appropriate administration route is therefore an important component of achieving optimal health outcomes and compassionate veterinary care.

Beyond selecting the appropriate devices, proper administration techniques are equally crucial for ensuring treatment success. The skill and expertise of veterinary professionals and caregivers significantly influence drug delivery outcomes [5, 22, 25, 26]. Injections, for instance, require precision to prevent complications such as tissue damage or infection. Newer technologies like microneedle patches also demand specialized application techniques and, in some cases, post-application monitoring to assess skin responses, drug absorption, or patch adherence, all of which contribute to achieving the desired therapeutic effect [1, 5, 22, 30–32]. As veterinary medicine advances, comprehensive training programs are fundamental in equipping professionals with the knowledge and skills required to administer both conventional and emerging drug delivery systems effectively.

Educating caregivers is also essential, particularly when they are responsible for administering treatments at home or on farms. Proper instruction in oral, topical, and injectable drug administration enhances treatment efficacy, reduces stress for animals, and minimizes veterinary costs [5, 21, 33, 34]. Incorrect administration can lead to ineffective treatment, increased discomfort, and higher veterinary expenses [6, 28, 34]. Ensuring that veterinary staff, pet owners, and caregivers are well-trained promotes better compliance, improved health outcomes, and a reduced need for veterinary interventions [33, 35].

**Fig. 1:**
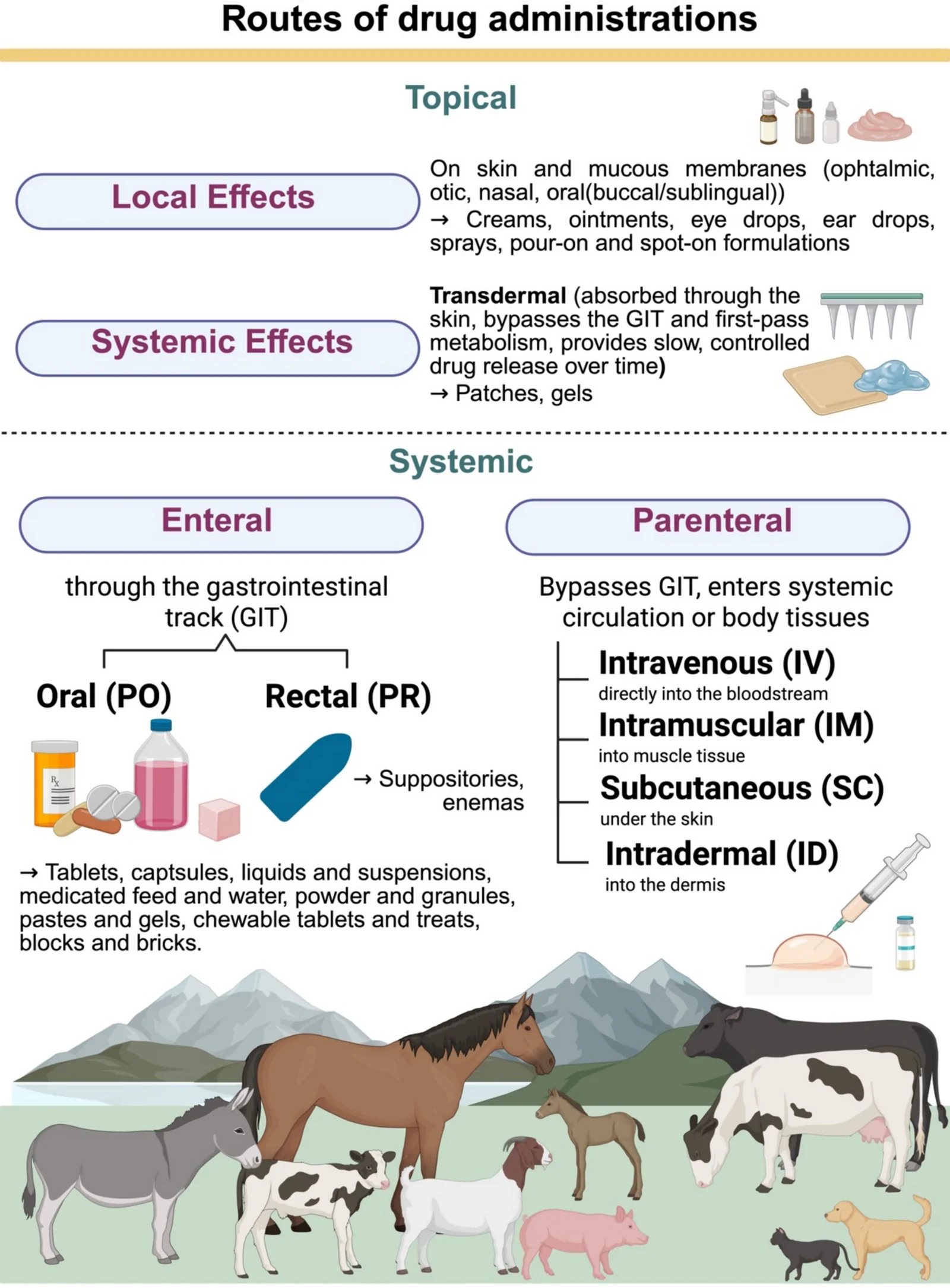
Routes of drug administration in veterinary medicine. Schematic representation of drug administration routes in veterinary medicine, categorized into enteral (oral and rectal), parenteral (intravenous, intramuscular, subcutaneous, and intradermal), and topical (local and systemic effects)

With these factors in mind, veterinary medicine incorporates three primary drug administration routes enteral, parenteral, and topical as shown in Fig. 1 each offering distinct advantages and challenges depending on the medication and the animal's needs.

### Enteral administration

Enteral administration involves delivering medications through the gastrointestinal tract (GIT) and is widely utilized in veterinary medicine due to its non-invasive nature, ease of administration, and suitability for long-term treatments [36, 37]. Among enteral routes, oral administration is the most commonly employed, as it aligns with natural feeding behaviors and generally minimizes stress for both animals and caregivers [5, 26, 37]. However, ensuring consistent drug absorption via this route can be difficult, as it is influenced by differences in gastrointestinal function, feeding habits, and overall physiology across species [37–40].

To improve compliance, a variety of pharmaceutical formulations have been developed, including liquid suspensions, flavored tablets, soft chews, and dissolvable films [41, 42]. Several orally administered veterinary products are widely used in clinical practice, including carprofen (Rimadyl®) for pain management in dogs [43], meloxicam (Metacam®) for analgesia in dogs and cats [44], and fenbendazole (Panacur®) as a broad-spectrum anthelmintic in livestock and companion animals [45]. These registered products illustrate the central role of oral delivery in veterinary pharmacotherapy. For larger animals such as cattle, horses, and sheep, boluses and medicated feeds are commonly employed to facilitate administration and ensure accurate dosing [5, 39, 46–48]. Intraruminal boluses are commonly administered using a balling gun, a device specifically designed to safely deliver large solid dosage forms into the rumen of cattle and small ruminants [49]. Intraruminal delivery is widely used in ruminants for the controlled release of antiparasitic agents, trace minerals, and therapeutic compounds via boluses placed in the rumen, enabling prolonged drug residence and sustained exposure [50]. In parallel, advancements in coating technologies enhance palatability by masking undesirable tastes, while specialized formulations are designed to improve drug release kinetics and stability during gastrointestinal transit [51–53]. For example, enteric coatings, such as those using hydroxypropyl methylcellulose phthalate (HPMCP) have been widely applied to protect active pharmaceutical ingredients from gastric degradation and enable targeted intestinal release [54–57]. Similarly, polymeric coatings like ethylcellulose and Eudragit® E are frequently used in veterinary formulations to support taste masking and site-specific delivery. For example, ethylcellulose has been utilized in dual-layered pellet systems designed for ruminants, enhancing rumen bypass and promoting controlled release in the abomasum; an approach that improves nutrient retention and minimizes premature degradation in the upper gastrointestinal tract [58]. Likewise, in companion animals, especially cats and dogs, palatability remains a critical determinant of treatment adherence. Hautala et al. developed flavored Eudragit® E coatings for feline minitablets using amino acid-based flavoring agents such as methionine, leucine, and thiamine hydrochloride. These formulations were shown to improve voluntary drug intake while maintaining desirable film properties, addressing the unique flavor sensitivity and behavioral characteristics of felines [59]. More broadly, formulation strategies like texture modification, dosage form selection, and incorporation of species-specific flavor profiles can significantly enhance acceptance in small domestic animals [60]. Evidence further supports the impact of palatability-enhancing approaches: Ahmed and Kasraian reported that the inclusion of appropriate flavoring increased voluntary drug intake in dogs from 20–40% to over 90%, highlighting the clinical value of such interventions [61]. Additionally, custom compounding services contribute to improved compliance by offering tailored dosage forms, including flavored suspensions, mini-tablets, capsules, and transdermal gels, adapted to the animal’s weight, taste preferences, and treatment regimen [62–64].

When voluntary oral ingestion is not feasible, feeding tubes and dosing syringes serve as essential tools for medication delivery [40, 65, 66]. These devices allow for accurate dosing and are particularly effective for administering liquid formulations to animals that are unwilling or unable to swallow medications [65, 66]. Selecting an appropriately sized device is critical to avoid discomfort or injury. For instance, nasoesophageal tubes typically range from 3.5 to 8 French (Fr), a unit that refers to the outer diameter of the tube, where 1 Fr equals 0.33 mm, while esophagostomy tubes are generally larger, often around 14 Fr or more, depending on the animal’s size and species [40, 65–67]. Improper sizing or poor tube design may cause irritation, stress, or even injury, potentially reducing compliance and compromising treatment outcomes [68]. Despite these challenges, these tools remain widely used due to their non-invasive nature, affordability, and adaptability across various veterinary applications.

While oral forms are the most common method, rectal administration serves as a valuable alternative when oral medication is not feasible, such as in animals experiencing vomiting or severe gastrointestinal disturbances [5, 25, 69]. Rectal administration has been widely studied for its effectiveness in emergency and therapeutic applications across various animal species. In small animals, rectal diazepam is a well-documented alternative for seizure management when intravenous access is unavailable, while levetiracetam has been shown to achieve rapid systemic absorption when administered rectally [70, 71]. In large animals, such as cattle and horses, rectal drug delivery can serve as a practical solution when oral administration is not possible due to gastrointestinal disturbances or handling difficulties. However, species-specific considerations must be addressed; for instance, in horses and cattle, rectal examinations pose a risk of rectal tears, necessitating careful administration [69]. Additionally, in pigs, rectal administration of certain antibiotics, such as tylosin, has been associated with adverse effects including edema of the rectal mucosa, anal protrusion, diarrhea, erythema, and pruritus; however, these side effects typically resolve uneventfully following discontinuation of the drug [72]. Despite its challenges, rectal administration remains a viable and often necessary route of drug delivery, offering both localized and systemic effects for treatments such as pain management, seizure control, and rectal inflammation, particularly when rapid absorption is required. Further details on additional enteral administration forms can be found in Table 1 and Fig. 1.

**Table 1:**
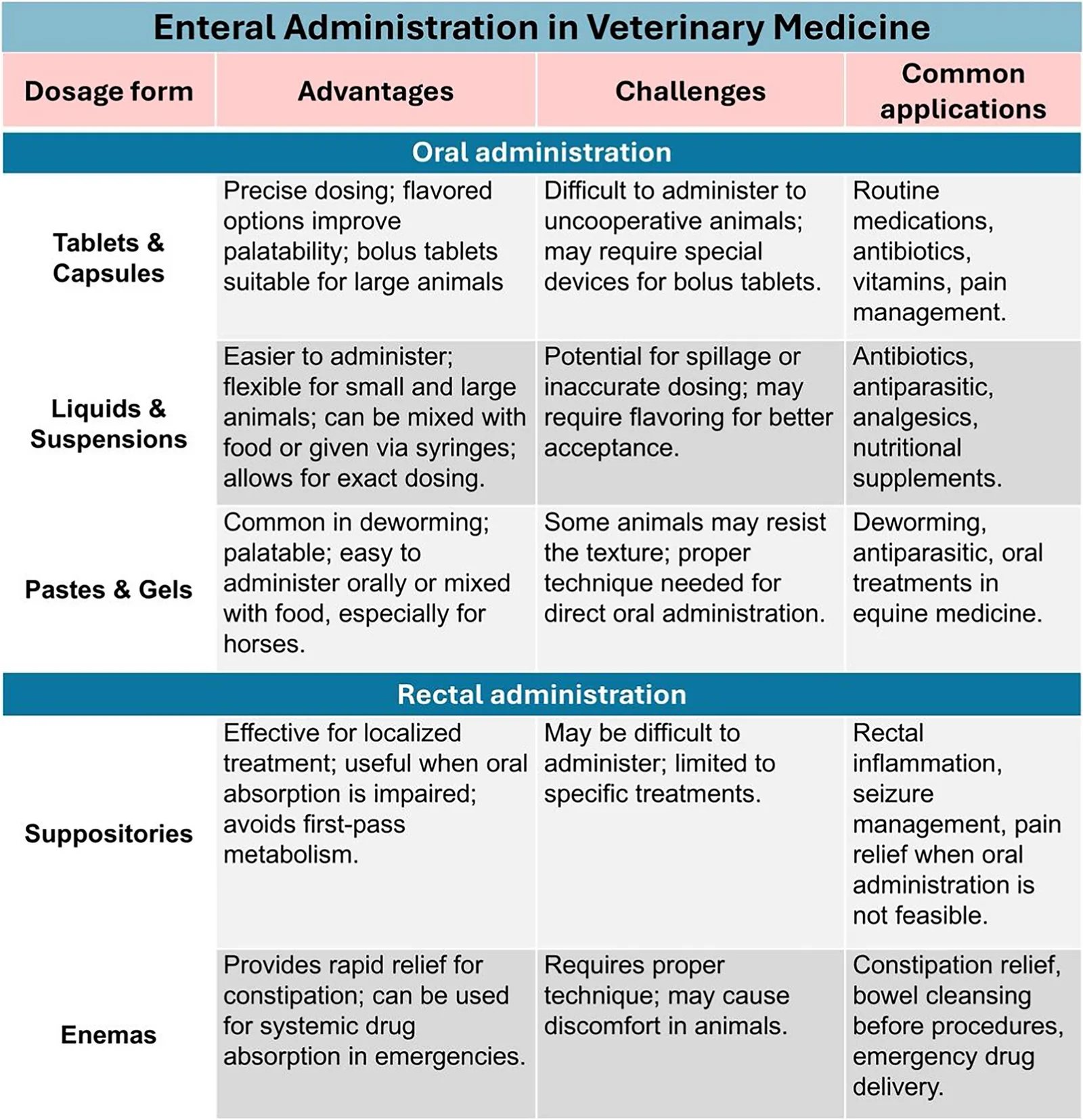
Enteral administration in veterinary medicine: dosage forms, advantages, challenges, and applications

### Parenteral administration

Parenteral methods involving injections are invaluable in veterinary medicine, offering rapid and reliable drug action crucial for acute or severe conditions [73]. These methods can be broadly categorized into four primary types based on the route of administration. Intravenous (IV) administration delivers medications directly into the bloodstream, providing the fastest onset of action, which is often critical during emergencies or when precise fluid and drug therapy is required [5, 73, 74]. Intramuscular (IM) injections, while slower in onset, allow for sustained drug release and are commonly used for vaccinations and long-acting treatments [25, 26, 73]. Subcutaneous (SC) injections, which are easier to administer, offer gradual absorption and are ideal for vaccines, fluid therapy, or long-term medications such as insulin or antibiotics [5, 25, 73]. Intradermal (ID) injections, administered into the dermis, are primarily used for diagnostic purposes, including allergy testing and tuberculosis screening, or for delivering specific vaccines [73]. Each of these routes serves distinct purposes and offers unique advantages, tailored to the specific needs of the animal and the clinical scenario at hand [25, 26, 73–76]. In addition to these classical injection routes, intramammary administration represents a specialized localized parenteral route in veterinary medicine and is a cornerstone of mastitis treatment in dairy cattle, enabling high local drug concentrations within the mammary gland while minimizing systemic exposure [77, 78].

Effective parenteral administration depends not only on the drug formulation but also on the compatibility of syringes and needles used for delivery [79]. These devices must be capable of handling various viscosities and volumes while ensuring accurate dosing and minimal risk of clogging [80, 81]. Additionally, needle design plays a crucial role in reducing tissue damage and improving injection efficiency [81]. A mismatch between the drug formulation and the device can lead to inefficiencies or complications, such as incomplete drug delivery or increased discomfort for the animal [82]. Despite these challenges, syringes and needles remain cost-effective and reliable tools, especially in urgent or acute treatments [80, 81]. However, they require technical proficiency to avoid complications such as infection, tissue trauma, or improper dosing [79, 82].

For instance, in domestic animals, IV administration is frequently used in emergencies to deliver fluids and drugs such as anesthetics or pain relievers during surgeries or after traumatic injuries [83]. IM injections are commonly used for administering medications like certain antibiotics (e.g., penicillin), corticosteroids, or melarsomine for treating heartworm infections in dogs [84]. SC injections are more frequently used for routine vaccinations, such as rabies and distemper, or for long-term treatments like insulin administration in diabetic pets and fluid therapy in dehydrated animals [85]. In contrast, farm animals such as cattle, horses, and sheep often require large-scale administration techniques. IV injections are used for critical interventions, such as treating severe infections with antibiotics or administering anti-inflammatory drugs [86]. IM injections are used for various therapeutic and preventive treatments, including some vaccines and vitamin injections. However, many livestock vaccines, such as the brucellosis vaccine in cattle, are now administered subcutaneously to minimize tissue damage and improve efficacy [84]. SC injections remain valuable in livestock due to their ease of use and are commonly employed for administering dewormers or vaccines. Although vaccines for foot-and-mouth disease (FMD) exist and are used in endemic regions, FMD vaccination is not currently practiced in the United States due to its FMD-free status [87, 88].

While parenteral administration is highly effective, it differs significantly from enteral routes in terms of invasiveness and immediacy of drug action. Unlike oral or rectal administration, which rely on gastrointestinal absorption, parenteral methods bypass the digestive system entirely, providing direct access to systemic circulation [5, 69, 75]. This distinction allows for faster therapeutic effects and eliminates concerns related to poor gastrointestinal absorption or first-pass metabolism. However, parenteral administration also requires technical skill, specialized equipment, and careful handling, often resulting in higher associated costs. Ensuring proper device compatibility and accurate administration techniques is essential to maximizing treatment success and minimizing risks. Moreover, appropriate training and safety precautions are essential, as parenteral administration can pose injury risks to operators, including needlestick injuries or harm during animal restraint. Further details on various parenteral administration methods can be found in Table 2.

**Table 2:**
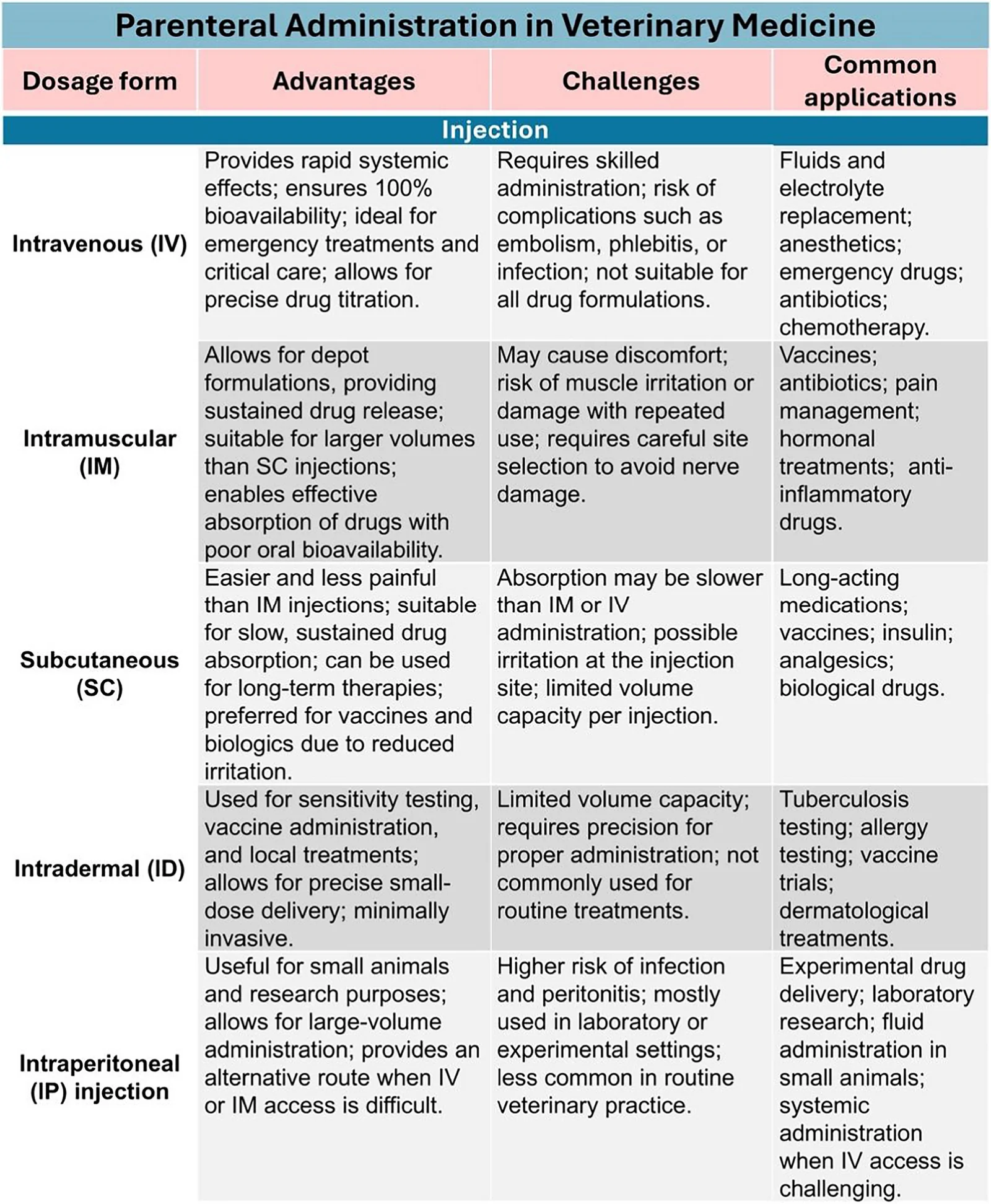
Parenteral administration in veterinary medicine: dosage forms, advantages, challenges, and applications

### Topical and transdermal administration of drugs and vaccines

Topical and transdermal administration methods are essential in veterinary medicine by offering both localized and systemic therapeutic effects. These approaches are particularly beneficial when targeting specific areas on the body or avoiding invasive methods like injections. While topical administration focuses on delivering drugs for localized effects, transdermal administration ensures systemic drug absorption through the skin. [89, 90].

Topical and transdermal drug delivery methods play a significant role in veterinary medicine by enabling both localized and systemic therapeutic effects without the need for invasive procedures. These approaches are particularly advantageous when repeated dosing, stress minimization, or targeted delivery is desired. The following subsection outlines the mechanisms, advantages, and applications of each method in veterinary contexts.

In the United States, veterinary transdermal products are regulated primarily by the U.S. Food and Drug Administration (FDA) through the Center for Veterinary Medicine (CVM) when they are classified as veterinary drugs [91, 92], whereas in Europe, veterinary medicinal products are regulated by the European Medicines Agency (EMA) in coordination with national competent authorities [93]. Certain topical antiparasitic products (e.g., flea and tick collars or spot-on ectoparasiticides) are regulated by the U.S. Environmental Protection Agency (EPA) when classified as pesticides [94]. In addition, some transdermal formulations may be prescribed under the U.S. Animal Medicinal Drug Use Clarification Act (AMDUCA), which permits extra-label use of approved human or veterinary drugs in animals under veterinary supervision when no suitable approved alternative exists [95].

To complement this discussion, Table 3 provides examples of transdermal veterinary products currently approved for clinical use, while Table 4 highlights promising transdermal formulations under investigation. These tables help illustrate the expanding role of transdermal delivery in veterinary therapeutics, from conventional treatments to novel drug development.

**Table 3:** Approved and compounded transdermal products for veterinary use

Name/Brand	Active Ingredient	Animal Species	Indication	Formulation Type	Approval/Reference
Recuvyra®	Fentanyl	Dogs	Postoperative pain	Transdermal solution	U.S. Food & Drug Administration (FDA) approved through the **Center for Veterinary Medicine (CVM)** 2014/Elanco Companion Animal Health
Seresto®	Imidacloprid + Flumethrin	Dogs, Cats	Flea and tick control	Polymer-based collar (slow-release system)	United States Environmental Protection Agency (EPA) as they fall under **pesticides**/Originally developed and marketed by Bayer Animal Health, but the brand is now owned by Elanco Animal Health 2020
Advantage Multi® (also marketed as Advocate®)	Moxidectin (flea adulticide) + Imidacloprid (antiparasitic)	Dogs, Cats	Parasitic control	Topical solution (Spot-on)	FDA-approved through the **Center for Veterinary Medicine (CVM)** 2007/Elanco
Mirataz®	**Mirtazapine**	Cats	**Management of weight loss** in cats with underlying chronic illness	Transdermal ointment	FDA-approved through the **Center for Veterinary Medicine (CVM)** 2018/Dechra Pharmaceuticals
Zorbium®	Buprenorphine	Cats	Postsurgical pain relief	Transdermal solution (topically applied to the inner surface of the cat’s ear)	FDA approved through the **Center for Veterinary Medicine (CVM)** 2022/Elanco
Banamine®	Flunixin meglumine	Cattle	Pain & fever from BRD, mastitis, foot rot	Pour-on solution	FDA approved through the **Center for Veterinary Medicine (CVM)** 2017/Merk First FDA-approved transdermal NSAID for food—producing animals
Centragard®	**Eprinomectin** (antiparasitic) + **Praziquantel** (tapeworm treatment)	Cats	Prevention of heartworm disease and treatment and control of roundworms, hookworms, and tapeworms	Transdermal solution (topical single-dose applicator)	FDA-approved through the **Center for Veterinary Medicine (CVM)** 2018/Boehringer Ingelheim Animal Health
Duragesic®	Fentanyl	Dogs, Cats, Horses	Postoperative pain Chronic pain management in cancer or orthopedic conditions	Transdermal patch	Permitted under the U.S. Animal Medicinal Drug Use Clarification Act (AMDUCA) Considered off-label or extra-label medication allowing veterinarians to prescribe them when no suitable approved alternative exists or when oral and injectable administration is impractical due too poor tolerance or stress Must be prescribed by a veterinarian, compounded by a licensed pharmacy, and used in accordance with AMDUCA regulations
*Compounded only*	Methimazole	Cats	Hyperthyroidism	Transdermal gel and cream, typically compounded in a PLO (pluronic lecithin organogel) base	
	Gabapentin		Neuropathic pain, anxiety, sedation		
	Prednisolone		Inflammatory and autoimmune disease		
*Compounded only*	Amitriptyline	Cats	Behavioral disorders, cystitis	Transdermal gel

**Table 4:** Transdermal platforms and products under investigation for veterinary applications

Platform/Product	Active Ingredient	Target Species	Indication	Formulation Type	Development Stage	Reference
Microneedle Patch (this work)	Meloxicam	Livestock	Postoperative and inflammatory pain	Dissolvable microneedle patch PVA–COL–CHI microneedles	Preclinical (in vitro/in vivo animal studies)	[96]
MN patch w/tilmicosin	Tilmicosin (antibiotic)	Sheep	Respiratory infection (e.g. *M. haemolytica*)	Polymeric microneedles	Preclinical	[97]
Transdermal ivermectin gel	Ivermectin	Cattle, pigs	Parasitic infections	Topical gel	In vitro/ex vivo	
Progesterone transdermal gel	Progesterone	Cows	Estrus synchronization/reproductive control	Bioadhesive gel	Early-stage trials	[98–100]
Transdermal enrofloxacin	Enrofloxacin (antibiotic)	Dogs, pigs	Systemic bacterial infection	Hydrogel patch	In vitro/ex vivo	[101]
Microneedle patch	Vaccines (rabies, influenza)	Dogs, livestock	Immunization	Dissolving microneedle patch	Preclinical animals (dogs, pigs)	[102]
Microneedle patch	Insulin	Dogs, Cats	Diabetes mellitus	Dissolving microneedle patch	Early preclinical	[103]
Nanoparticle-loaded patch	Carprofen	Dogs	Chronic pain	Nanocarrier enhanced patch	Experimental (in-vitro and modeling)	[104]
Compounded Transdermal gel	Gabapentin	Cats	Chronic pain, anxiety	PLO-based transdermal gel	Limited absorption; variable results. Systemic levels inconsistent; mixed clinical outcomes	[105]
	Tramadol		Moderate to severe pain		Low absorption in cats	[106]
	Ketoprofen		Inflammation and musculoskeletal pain		Limited preclinical data	
Commercial patch (off-label use)	Lidocaine	Dogs	Localized pain, neuropathic conditions	Transdermal patch	Off-label veterinary use. Provides localized analgesia with minimal systemic exposure	[107]

#### Topical administration

Topical administration involves applying drugs directly to the skin or mucous membranes to treat localized conditions [108, 109]. These formulations are ideal for managing infections, inflammation, wounds, and other dermatological issues without systemic drug involvement [110, 111]. Creams and ointments are widely used for treating bacterial or fungal infections, inflammation, or minor wounds [112, 113]. For example, antibiotic ointments are commonly applied to surface infections in dogs, while anti-inflammatory creams may be prescribed to manage skin irritation in horses [114, 115] These formulations are often combined with systemic medications to enhance therapeutic outcomes.

Spot-on treatments, frequently used for parasite control, involve applying a small amount of liquid to a specific area, such as the base of the neck in dogs or along the back in cattle [116–118]. These treatments are highly effective against fleas, ticks, and other external parasites, offering a convenient and stress-free solution for animals. Similarly, pour-on treatments, primarily used in livestock such as cattle and sheep, are applied along the back, allowing the medication to spread across the skin for broad coverage against parasites like lice and flies [119].

Other specialized topical dosage forms include ophthalmic preparations and otic (ear) drops, each designed to treat localized conditions while minimizing discomfort and promoting consistent treatment adherence [1]. Ophthalmic formulations, such as sterile drops, ointments, and gels, are essential for managing eye conditions like infections, inflammation, or trauma, providing targeted therapy with reduced risk of systemic side effects [120]. For example, antibiotic drops are frequently used to treat bacterial conjunctivitis in dogs, while anti-inflammatory gels may be prescribed for conditions such as equine uveitis [121–123]. Ensuring appropriate viscosity, pH, and sterility is critical in these formulations to improve ease of application and reduce the risk of secondary infections.

Otic drops are specifically designed for ear infections and other otic conditions, penetrating the ear canal effectively to target pathogens such as bacteria or fungi [124]. Common applications include treating otitis externa in dogs or mite infestations in cats [125]. Many otic formulations combine antibacterial, antifungal, and anti-inflammatory agents to provide comprehensive care, ensuring quick relief and reducing infection recurrence [125].

Inhalants constitute a distinct class of veterinary drug delivery systems designed to manage respiratory conditions such as airway inflammation, asthma, and bronchitis, particularly in cats, horses, and small animals**.** These formulations, delivered via metered-dose inhalers, dry powder inhalers, or nebulized solutions, offer the advantage of rapid onset and localized drug action, thereby reducing systemic exposure and side effects [126]. Inhalant devices work by converting medications into fine mists for deep pulmonary deposition, enabling efficient absorption directly at the site of inflammation. Commonly administered agents include bronchodilators and corticosteroids, which are frequently used to treat chronic conditions like feline asthma and recurrent airway obstruction (RAO) in horses [127–129]. Inhalants offer a non-invasive way to deliver high drug concentrations directly to the lungs, enabling targeted and effective respiratory therapy [126].

Specialized dosage forms such as ophthalmic preparations, otic drops, and inhalants play a crucial role in veterinary care by offering targeted, localized treatments for specific conditions. Ophthalmic and otic formulations minimize systemic exposure while improving treatment efficacy, whereas inhalants provide an effective, non-invasive approach for managing chronic respiratory diseases. However, these treatments also present challenges, including difficulties in administration, particularly in uncooperative animals, and the need for specialized equipment such as nebulizers, which may not always be readily available in all veterinary settings [126, 130].

#### Transdermal administration

Transdermal administration provides systemic effects by enabling drugs to penetrate the skin barrier and enter systemic circulation [131, 132]. This method is particularly advantageous for animals requiring consistent drug levels in plasma over extended periods or for medications with poor oral bioavailability [133, 134]. One common approach is the use of transdermal patches, which deliver drugs at a controlled rate over time [135]. For instance, fentanyl patches are widely used for postoperative and chronic pain management in dogs and cats, offering continuous analgesia for up to 72 h. While transdermal drug-delivery systems remain relatively limited in veterinary applications for livestock, heat detection patches are widely used in cattle for behavioral estrus monitoring, not drug delivery. These pressure-sensitive patches are applied to the tailhead and change color or appearance when the animal is mounted, helping farmers accurately time artificial insemination. For hormonal synchronization, intravaginal progesterone-releasing devices such as CIDRs are more commonly used in cattle [10, 100, 136, 137].

The success of transdermal patches depends on effective adhesion, drug permeation, and animal tolerance. These factors are influenced by species-specific skin characteristics, including thickness, lipid composition, hydration level, and the presence of fur or hair, all of which can complicate both patch attachment and drug absorption [3, 138–140]. Inadequate adhesion may lead to subtherapeutic dosing, while repeated application or occlusion can trigger skin irritation or allergic dermatitis [3, 141, 142].

To address these challenges, many veterinary transdermal formulations incorporate permeability enhancers, which temporarily alter the structure of the stratum corneum to facilitate drug penetration. Common enhancers include fatty acids (e.g., oleic acid), alcohols (e.g., ethanol), surfactants, terpenes, and solvents like dimethyl sulfoxide (DMSO) [143–145]. These agents function by disrupting lipid bilayers, enhancing drug solubility, or fluidizing skin lipids to facilitate diffusion through the skin barrier [143, 144]. However, their concentrations must be carefully optimized, as excessive permeability can result in local irritation or systemic toxicity, particularly with chronic use [143, 145].

Importantly, not all drugs are suitable for transdermal delivery. The stratum corneum presents a significant diffusion barrier, and only molecules with appropriate physicochemical properties, such as low molecular weight (typically under 500 Daltons), moderate lipophilicity, and sufficient aqueous solubility, can effectively permeate the skin [146, 147]. These constraints limit the range of drugs amenable to transdermal administration in veterinary medicine.

Despite these challenges, transdermal patches remain a valuable, non-invasive option for long-term therapy, reducing the need for frequent dosing and minimizing stress from repeated injections [148, 149]. Ongoing advancements in formulation technologies, such as the inclusion of permeability enhancers, continue to expand their applicability and effectiveness across a range of animal species.

Beyond patches, transdermal gels, including hydrogels and organogels, offer effective means of drug delivery with both localized and systemic effects. Hydrogels, which are water-based and highly hydrophilic, enhance skin hydration and permeability, making them particularly suitable for hydrophilic drugs such as antibiotics, analgesics, and anti-inflammatory agents [150, 151]. For example, silver sulfadiazine hydrogel is commonly used for burn wound care in dogs and cats due to its antimicrobial and soothing properties [152–154]. Diclofenac sodium and ketoprofen have also been formulated into hydrogels for topical anti-inflammatory treatment in horses and companion animals [155–158]. Their moisture-retaining properties also provide a cooling and soothing effect, making them ideal for wound care and burn treatments [150, 159]. In contrast, organogels, which use organic solvents or oils as a base, are particularly effective for lipophilic (fat-soluble) drugs, such as steroids, local anesthetics, and antifungal agents [151, 160]. For instance, isoflupredone acetate, a corticosteroid used for equine joint inflammation, has been formulated into oil-based gels for transdermal use [161]. Additionally, miconazole organogels are used in treating canine and feline dermatophytosis, offering enhanced absorption into the skin’s lipid layers [162]. These formulations enhance drug absorption, making them an effective option for skin disorders, localized pain management, and infections, particularly when ease of application and reduced stress are priorities in veterinary care [163].

Another innovative transdermal method is the use of microneedle patches, which employ micro-sized needles to create channels in the skin, facilitating direct drug delivery into dermal layers with minimal discomfort [164–166]. These patches can be biodegradable, dissolving after drug release, or non-biodegradable, requiring removal after use [32, 167]. While microneedle patches hold great promise for vaccinations and systemic drug delivery for large molecules, their effectiveness depends on proper penetration without causing harm to the animal [14, 168]. Additionally, ensuring ease of use is essential, particularly for veterinary practitioners, caregivers, or pet owners who may not have specialized training. Although microneedle technology is still emerging, its potential for efficient, direct drug delivery with fewer risks of complications makes it a promising advancement in veterinary medicine [166, 169, 170].

Overall, transdermal drug delivery methods, including patches, gels, and microneedle technologies, offer promising non-invasive alternatives for veterinary medicine, balancing controlled drug release with improved compliance, though further research is needed to optimize adhesion, absorption, and ease of administration for diverse animal species.

## Advances in veterinary transdermal drug and vaccine delivery: mechanisms and emerging technologies

**Fig. 2:**
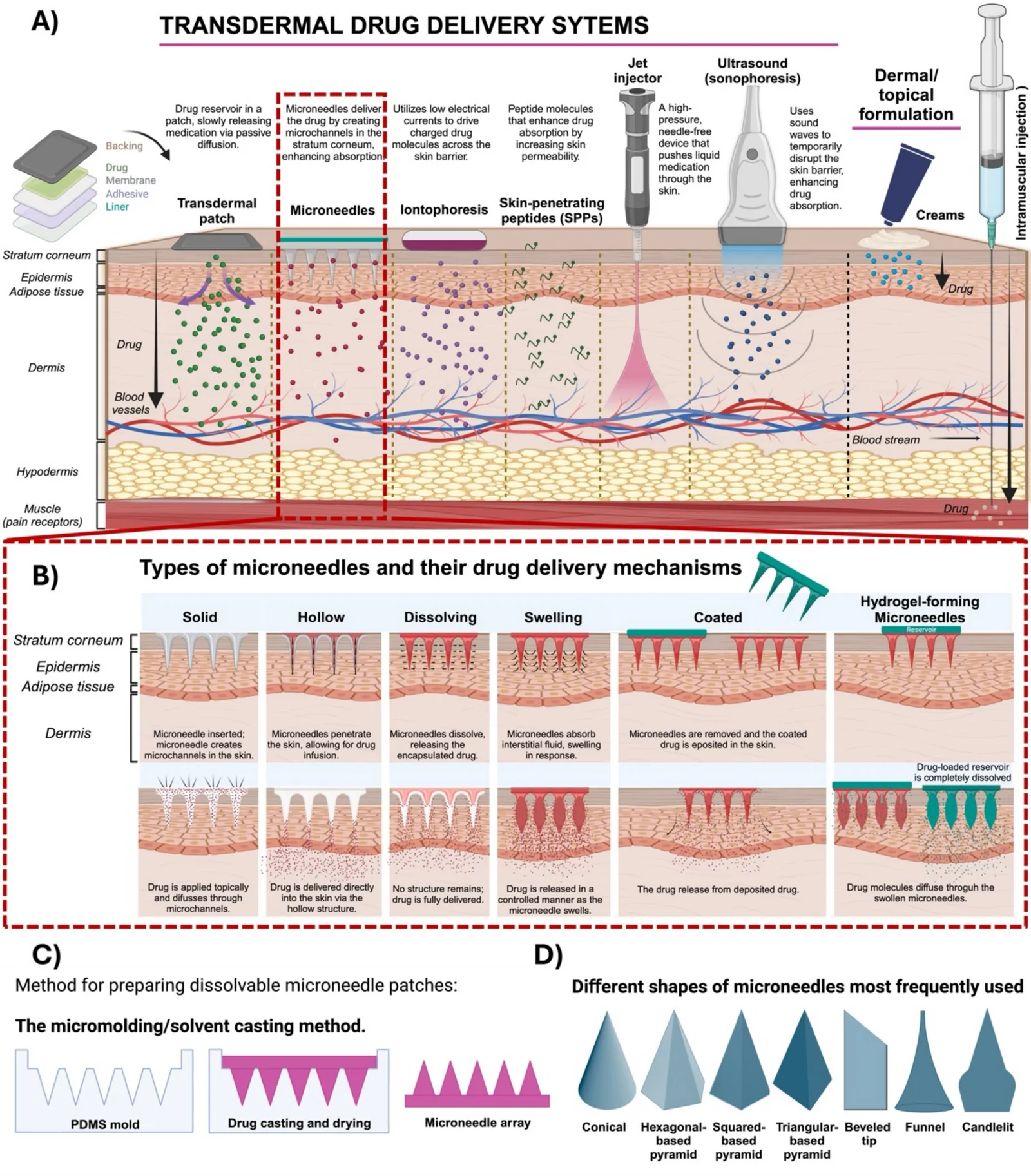
Overview of transdermal drug delivery systems and microneedle-based drug administration. This figure illustrates various transdermal drug delivery systems, including patches, microneedles, iontophoresis, sonophoresis, and jet injectors, along with their mechanisms for enhancing drug absorption through the skin. A detailed section highlights different types of microneedles solid, hollow, dissolving, swelling, coated, and hydrogel-forming depicting their unique drug delivery mechanisms. Additionally, common microneedle shapes and the micro molding/solvent casting method for fabricating dissolvable microneedle patches are presented

While transdermal drug delivery presents challenges related to skin permeability and drug absorption, continuous advancements in pharmaceutical sciences have led to innovative strategies that enhance its efficacy and broaden its clinical applications [171, 172]. Understanding the mechanisms governing transdermal absorption is fundamental to optimizing drug formulations and delivery methods. Various transdermal systems (Fig. 2), including passive diffusion-based patches, microneedle-assisted technologies, and nanocarrier-based formulations, have been developed to overcome absorption barriers and improve therapeutic outcomes [169, 173]. Microneedle-based delivery has gained significant attention due to its ability to bypass the stratum corneum and facilitate controlled drug release, minimizing systemic side effects [169]. Additionally, nanocarrier-based formulations, such as invasomes, have demonstrated enhanced permeation through the skin by modifying lipid interactions and increasing drug solubility [173]. Furthermore, emerging techniques such as electroporation and ultrasound-assisted drug penetration are being explored to enhance transdermal drug transport [174, 175]. Electroporation, which transiently disrupts cell membranes through short electrical pulses, has shown promise in improving macromolecule delivery across the skin barrier [174]. Similarly, low frequency sonophoresis enhances transdermal transport by temporarily altering lipid bilayers through cavitation effects, providing a non-invasive alternative for controlled drug administration [175]. These advancements highlight the growing potential of transdermal technologies in achieving efficient and patient-friendly drug delivery.

As veterinary medicine continues to develop, alternative drug delivery systems have gained importance in improving treatment outcomes [1]. Transdermal drug delivery (TDDS) provides a direct route for drug absorption through the skin, allowing for controlled and sustained drug release [12, 133]. Unlike traditional oral or injectable routes, which are prone to fluctuations in drug levels, TDDS offers a steady release profile that helps maintain stable plasma concentrations over time—a benefit particularly important for drugs with narrow therapeutic windows [176]. This stability is particularly beneficial for drugs with narrow therapeutic windows, as it minimizes the risks associated with peak-trough variations [131]. By eliminating the need for gastrointestinal absorption, transdermal delivery minimizes degradation issues associated with enzymatic metabolism, improving the consistency and efficiency of drug therapy [131, 177]. However, while skin permeability presents formulation challenges, technologies such as penetration enhancers and microneedle-assisted delivery are being developed to overcome these barriers [176, 178]. These innovations continue to expand the role of TDDS in veterinary medicine, offering a reliable and patient-friendly alternative to conventional delivery methods.

The concept of transdermal drug delivery has historical roots in ancient civilizations, where medicinal substances were applied topically using poultices, plasters, and salves made from herbs, clay, and plant extracts [179]. Although the mechanisms of absorption were not yet understood, these practices laid the foundation for modern transdermal technologies. The first scientifically developed transdermal patches emerged in the 1970 s, with the FDA approving the scopolamine patch for motion sickness in 1979. Subsequently, in 1981, nitroglycerin patches were introduced for angina treatment, and in 1991, nicotine patches became available for smoking cessation [180–182]. Over the years, advancements in materials, drug formulations, and engineering have expanded TDDS applications to include pain management, hormone therapy, and vaccine administration.

Despite the advantages offered by transdermal drug delivery, its effectiveness is often limited by the skin’s natural barrier function. The skin's primary function is to protect the body from external substances, making drug permeation particularly challenging [183]. The stratum corneum, the outermost layer of the epidermis, presents a formidable barrier, limiting the penetration of most drugs, especially those with high molecular weight and hydrophilicity [90, 177, 179, 184, 185]. Fundamentally, transdermal drug delivery relies on the ability of drugs to penetrate this protective barrier and reach systemic circulation. Given the skin’s inherent resistance to foreign substances, specialized delivery mechanisms are required to enhance drug permeation.

### Mechanisms

**Fig. 3:**
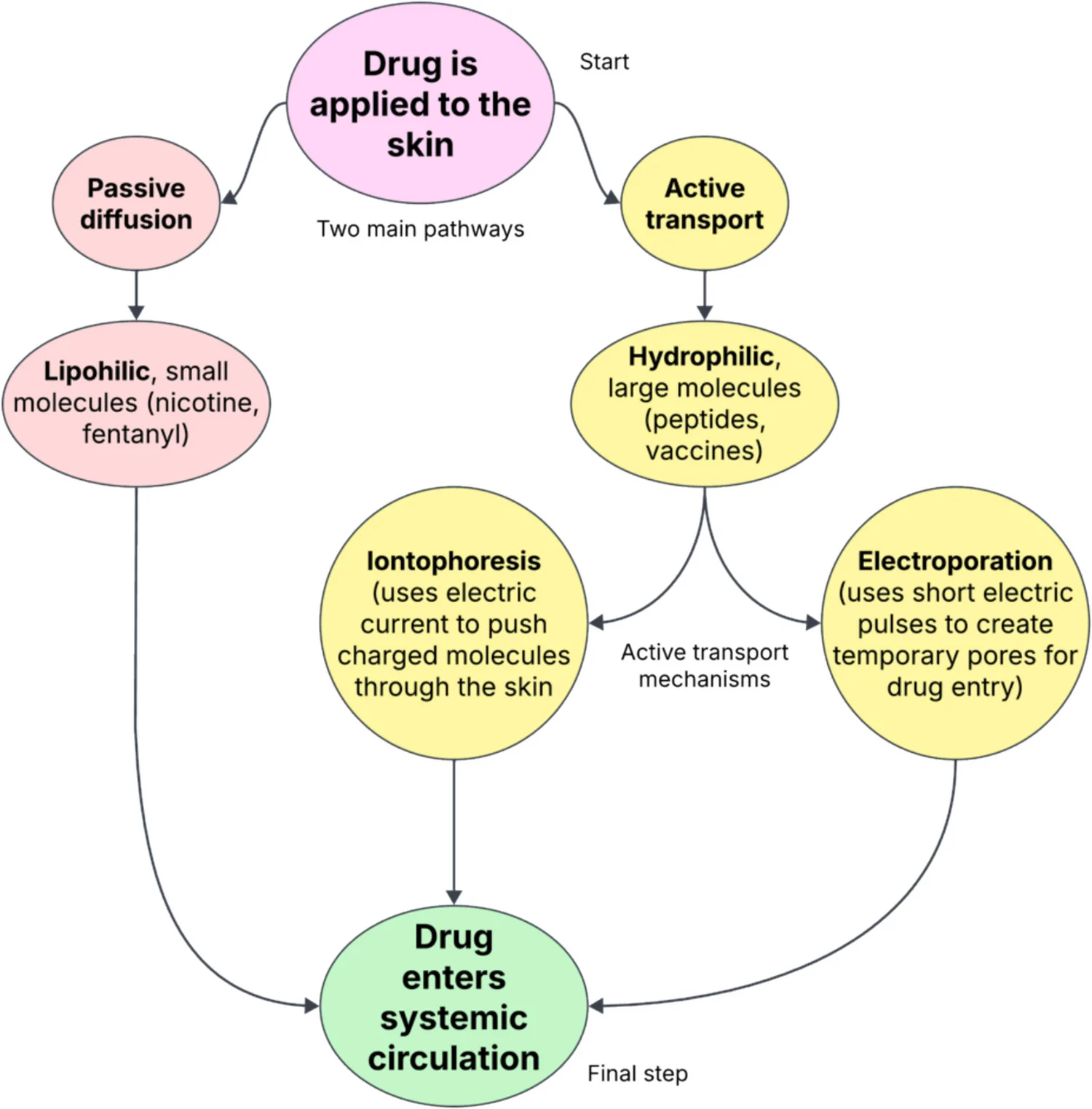
Mechanisms of transdermal drug delivery. Pathways of transdermal drug delivery, including passive diffusion for lipophilic molecules and active transport mechanisms like iontophoresis and electroporation for hydrophilic drugs

Various strategies have been researched to optimize transdermal drug absorption, broadly categorized into passive diffusion-based absorption and active transport-enhanced systems, as represented in Fig 3. Passive diffusion exploits the natural physicochemical properties of drugs to cross the skin barrier, while active transport mechanisms, such as microneedles, iontophoresis, and sonophoresis, facilitate enhanced drug penetration by temporarily disrupting or bypassing the stratum corneum [180, 186].

### Passive diffusion

Passive diffusion is widely recognized as the primary mechanism of drug absorption in transdermal delivery systems, enabling molecules to migrate from a region of higher concentration such as a drug reservoir or patch into the skin and eventually the bloodstream without requiring external energy or active transport mechanisms [131, 171]. This process is predominantly driven by a concentration gradient and is influenced by the physicochemical properties of the drug, including molecular weight and lipophilicity [187, 188].

The efficiency of passive diffusion in transdermal drug delivery is largely dependent on the structural composition of the skin, particularly the stratum corneum, the outermost layer composed of keratinized cells that form a protective barrier [171, 189]. Due to its low permeability, only certain drugs, typically small, moderately lipophilic (fat-soluble) molecules can effectively diffuse through this layer [190, 191]. Veterinary transdermal applications that meet these criteria include fentanyl (for analgesia), estradiol (for hormonal regulation), and methimazole (for managing feline hyperthyroidism) [192–195]. Once past the stratum corneum, the drug reaches the viable epidermis and dermis, where it encounters blood vessels and is subsequently absorbed into systemic circulation [196]. The rate of diffusion is influenced by multiple factors, including the concentration gradient, molecular size, lipophilicity, and the skin’s hydration level and thickness [197].

Despite its limitations, passive diffusion remains the foundation of transdermal drug delivery systems in both human and veterinary medicine [198]. In human healthcare, widely adopted transdermal products such as nicotine patches, hormone replacement therapy (HRT) products, fentanyl patches, and contraceptive patches have demonstrated the effectiveness of delivering drugs through the skin for systemic therapeutic effects. For instance, nicotine patches help reduce withdrawal symptoms and improve smoking cessation outcomes, while HRT patches provide controlled delivery of estrogen or testosterone with reduced hepatic side effects [199, 200]. Likewise, transdermal fentanyl has shown comparable analgesic efficacy to sustained-release morphine in chronic pain management, with fewer side effects such as sedation or constipation [201]. Additionally, contraceptive patches offer effective ovulation suppression while improving adherence through weekly dosing schedules, making them a more convenient alternative to daily oral contraceptives [202].

The success of these human therapies has significantly influenced the development of transdermal systems in veterinary medicine, informing drug selection, formulation design, and dosing strategies across species. For example, fentanyl patches are commonly used in dogs and cats for postoperative and chronic pain, providing continuous analgesia for up to 72 h [203–205]. Estradiol patches are used in livestock to regulate reproductive hormones and facilitate estrus synchronization [99]. In small animals, transdermal methimazole gels offer an alternative to oral tablets for treating feline hyperthyroidism, especially in patients that resist pill administration [192, 193].

Nevertheless, passive diffusion-based systems are still less widely used in veterinary medicine due to species-specific variations in skin permeability. Factors such as skin thickness, lipid content, fur density, and grooming behaviors can significantly impact drug absorption and patch adherence. Despite these challenges, fentanyl patches have been successfully utilized in dogs, cats, and horses, providing prolonged analgesia without the need for repeated injections [10, 137, 206, 207]. Methimazole transdermal gels are used to treat hyperthyroidism in cats, offering a practical alternative for animals that are difficult to medicate orally [208].

More recently, in 2022, the FDA approved the first transdermal formulation of buprenorphine (Zorbium®) for post-surgical pain control in cats, representing a significant milestone in the development of veterinary-specific transdermal therapies [209] While transdermal delivery in veterinary settings is not yet as widespread as in human medicine, ongoing research and technological innovations, such as enhanced permeability systems, optimized adhesive materials, and species-specific formulations, are steadily expanding its therapeutic potential.

### Active transport

Active transport is a key strategy in advanced transdermal systems, enhancing drug absorption when molecules are too large or hydrophilic (water-soluble) to penetrate the skin via passive diffusion alone [210]. Unlike passive methods, active transport utilizes external forces to temporarily disrupt the skin barrier, facilitating drug penetration [210, 211]. Two widely used techniques in transdermal drug delivery are iontophoresis and electroporation.

Iontophoresis utilizes a low electrical current to facilitate the transdermal delivery of charged molecules by generating electrostatic forces that drive their movement across the skin. This process operates through two primary mechanisms: electro repulsion (electromigration), where the applied electric field directly propels charged molecules through the skin, and electroosmosis, which induces solvent flow due to the electric field, assisting in the transport of both ionic and neutral molecules. This technique is especially effective for delivering ionic drugs and small peptides that would otherwise have limited skin permeability [212, 213]. This method has been applied in clinical settings for the delivery of medications such as lidocaine for local anesthesia and anti-inflammatory drugs for musculoskeletal conditions [214]. Electroporation, in contrast, applies short, high-voltage pulses to create temporary micropores in the stratum corneum, momentarily disrupting the skin’s protective barrier [215]. This approach enables the delivery of larger molecules, including biologics such as proteins and vaccines, making it a promising strategy for drugs that cannot diffuse naturally due to their size or charge [216, 217]. Recent studies have reported the development of hydrogel electronic devices capable of transdermal delivery of nucleic acids, showing potential for in vivo applications [218].

The application of active transport in transdermal drug delivery has proven to be a beneficial approach in both human and veterinary medicine. Iontophoresis has been successfully applied to enhance corticosteroid delivery, improving treatment outcomes for localized inflammation, pain, and conditions such as arthritis [214, 219, 220]. It is also used for transdermal administration of local anesthetics, providing needle-free pain relief during minor surgical procedures [175, 219, 221]. Additionally, iontophoresis facilitates the delivery of peptides and proteins, including insulin, offering a potential alternative to injections for diabetes management [222, 223].

Meanwhile, electrochemotherapy (ECT), which leverages electroporation to improve chemotherapeutic drug uptake by tumor cells, has shown promise in cancer treatment, enhancing drug absorption while minimizing systemic side effects [224, 225]. Studies have demonstrated its efficacy in treating cutaneous and deep-seated tumors, including hepatic and pancreatic cancers, with response rates reaching 84% in some cases [226]. Likewise, ETC preserves critical structures, making it a viable option for tumors near blood vessels and nerves [224]. On the other hand, ECT is effectively utilized in veterinary oncology to treat various tumors in companion animals and equine patients. Studies have demonstrated its efficacy in managing cutaneous and subcutaneous tumors in dogs and cats, including mast cell tumors, squamous cell carcinomas, and soft tissue sarcomas, while in equine patients, ETC has shown promise in treating sarcoid and squamous cell carcinomas [227–229].

As research continues to refine active transport techniques, these innovations hold great promise for expanding transdermal drug delivery applications, particularly for biologics, vaccines, and protein-based therapies in both human and veterinary medicine.

Both of these techniques aim to increase the permeability of the skin to drugs, enabling more effective and faster absorption compared to passive diffusion alone, especially for drugs that are otherwise difficult to deliver via transdermal systems.

#### Emerging technologies in transdermal drug and vaccine delivery systems

As previously discussed, transdermal patches are among the most widely used systems for delivering drugs through the skin, offering both localized and systemic effects [230]. These patches contain the active drug in a carrier material, allowing for controlled drug release over extended periods. Depending on their design, transdermal patches can regulate drug diffusion rates, optimize bioavailability, and improve patient compliance by minimizing frequent dosing and invasive administration [9, 135, 182]. Several advanced patch designs have been developed, each with distinct mechanisms to enhance drug permeation and therapeutic efficacy [9].

Reservoir patches, which hold the active drug in a liquid or gel reservoir separated from the skin by a rate-controlling membrane, provide precise and sustained drug release, making them ideal for potent drugs with narrow therapeutic windows [199, 231, 232]. In veterinary medicine, this design has been applied in fentanyl, lidocaine, and buprenorphine patches, which are used in dogs, cats, and horses for postoperative and chronic pain management [107, 233–235]. Fentanyl transdermal patches (e.g., Duragesic®) are widely used in small animals and horses, particularly for visceral or surgical pain, offering long-lasting analgesia with stable plasma concentrations [233, 236, 237]. Zorbium®, a recently FDA-approved buprenorphine transdermal solution for cats, delivers sustained opioid therapy following surgery, reducing the need for repeated injections during recovery [209, 238]. Lidocaine patches, although originally developed for humans with neuropathic pain, are safely used in dogs and cats due to minimal systemic absorption and high local tissue concentrations, which allow for effective localized pain relief with low systemic risk [107, 237]. These transdermal systems improve both the efficacy and practicality of veterinary pain management, particularly in cases where consistent plasma drug levels and reduced handling are critical.

In addition to reservoir systems, matrix patches, which embed the active drug directly into a polymeric layer, have gained traction in veterinary medicine due to their simplified structure and sustained release capabilities [199, 232, 239]. Recent pharmacokinetic studies in horses have shown that matrix-type fentanyl and buprenorphine patches can maintain therapeutic plasma concentrations for up to 96 h, particularly when applied to anatomical sites like the ventral tail base, which produced more consistent drug absorption compared to more mobile areas such as the inguinal or metacarpal regions [240, 241]. Beyond pharmacokinetics, studies have demonstrated that mechanical factors, including skin mobility, animal grooming behavior, fur density, and the method used to secure the patch (e.g., direct adhesion versus bandaging), can significantly affect both patch adherence and drug delivery efficiency [241–243]. For example, one study found that patches affixed using different attachment methods showed variation in drug depletion rates, indicating that even with the same formulation, the extent of skin contact, and mechanical stability can influence how much drug is absorbed systemically [241, 242]. These findings underscore the importance of optimizing not only the formulation and drug selection, but also application technique and anatomical placement, to ensure consistent and effective analgesia across veterinary species.

Building upon these foundational systems, multilayer patches have been developed to enhance delivery efficiency and drug stability through the incorporation of multiple functional layers. Multilayer patches enhance drug delivery efficiency by incorporating multiple functional layers, each playing a specific role. A typical multilayer patch may consist of a drug reservoir layer, an adhesive layer for skin attachment, and a protective backing to prevent drug degradation and leakage [244, 245]. By optimizing drug diffusion through sequential layers, multilayer patches allow for tailored drug release profiles, making them ideal for pain management, hormone therapy, and nicotine replacement treatments in the case of human use for example [11, 149, 246–249]. While these systems improve drug stability and absorption, their complex structure can pose manufacturing challenges, limiting widespread adoption in veterinary medicine [250].

To address some of these limitations and further enhance transdermal efficiency, recent advancements have focused on next-generation platforms such as microneedle patches, a minimally invasive and highly efficient drug delivery system [164, 165, 251]. Unlike conventional patches, they utilize microscopic needles to bypass the stratum corneum, creating temporary microchannels that facilitate rapid and controlled drug absorption while remaining virtually painless [165]. This feature not only improves bioavailability but also reduces variability in drug absorption, making it a more consistent and reliable alternative to traditional systems.

Microneedle patches have demonstrated significant potential in biologic therapies, including vaccinations, insulin administration, and localized pain management [168, 252–258]. Their ability to deliver larger molecules, such as proteins, peptides, and nucleic acids, sets them apart from conventional transdermal systems, which are largely limited to small, lipophilic compounds [168]. Additionally, microneedle patches can be designed for self-administration, reducing the need for trained personnel and improving patient compliance, particularly in remote veterinary settings where frequent handling of animals may be challenging [259].

Beyond drug delivery, microneedle technology has expanded beyond drug delivery into diagnostic and therapeutic applications, offering minimally invasive solutions for real-time health monitoring and enhanced vaccine efficacy [260]. In diagnostics, microneedles enable interstitial fluid (ISF) sampling, providing an alternative to blood collection for biomarker analysis [260, 261]. These devices facilitate continuous monitoring of proteins and metabolites, improving early disease detection and personalized medicine. Therapeutically, microneedles enhance vaccine delivery by targeting the skin’s antigen-presenting cells, leading to stronger immune responses with reduced doses [262, 263]. Dissolvable microneedle patches further improve vaccine stability and controlled antigen release [264]. These systems are typically fabricated from biocompatible polymers that degrade safely within the skin. In our work, we have developed biodegradable multicomponent microneedle patches composed of PVA, collagen, and chitosan for the delivery of meloxicam, demonstrating their effectiveness for sustained transdermal drug release in veterinary applications. These patches dissolve within hours of application, eliminating the need for removal and reducing sharps waste, an important advantage for both clinical and field use [96]. With continuous advancements in material science, formulation strategies, and delivery mechanisms, microneedle patches are poised to address many of the limitations of traditional patch systems, making them a promising alternative for a wide range of therapeutic applications.

## Innovative alternatives in transdermal drug and vaccine delivery: microneedle patches

Microneedle technology has advanced significantly since its initial conceptualization. Originally proposed by Gerstel and Place in 1976 for transdermal drug delivery, its practical application was first demonstrated in 1998 by Dr. Mark Prausnitz and his team at Georgia Tech, who showed that microneedles could create microchannels in the skin with minimal disruption, enabling efficient and less invasive drug administration [265–267]. These early studies laid the foundation for rapid innovation in microneedle design and materials, leading to expanding applications across drug delivery, medical and pharmaceutical fields in human medicine and veterinary therapeutics [268, 269].

Microneedle patches have innovated transdermal drug delivery by creating controlled microchannels in the stratum corneum, facilitating efficient drug absorption while remaining painless and minimally invasive. These microneedles, typically 100 to 1000 µm in length and a few micrometers in diameter, are designed to penetrate the outer skin layers without stimulating nerve endings [167, 251]. This size range allows them to reach the epidermis and upper dermis while avoiding deeper penetration that could activate pain receptors [270]. Studies have shown that shorter microneedles, such as those 480 μm in length, cause significantly less pain compared to longer ones, as they do not reach the deeper dermal layers where nerve endings are more prevalent [270, 271]. While these findings are primarily based on human studies, species-specific variations in skin anatomy and nerve distribution suggest that pain perception and tolerability of microneedle patches may differ among animal species. Therefore, further research is needed to optimize microneedle design and application protocols tailored to the anatomical and physiological characteristics of each target species.

Additionally, microneedles with a tip diameter smaller than 15 μm are essential for controlled and less painful skin penetration [272]. Their structural geometry, including conical, pyramidal, cylindrical, and beveled designs, significantly influences penetration depth, drug release kinetics, and mechanical durability [272–274]. Recent advancements have introduced hexagonal, triangular-based pyramidal, funnel-shaped, and candlelit microneedles, each tailored to optimize mechanical strength, drug diffusion rates, and skin permeability [275–278]. For instance, hexagonal and triangular-based microneedles enhance uniform penetration, while funnel and candlelit structures improve drug flow and retention at the application site [278].

Microneedles are classified based on their composition and drug delivery mechanism [279]. Solid microneedles create temporary microchannels for passive drug diffusion, whereas coated microneedles have drug layers that dissolve upon insertion, delivering medication directly into the dermis [271, 272]. Dissolvable microneedles, composed of biodegradable polymers such as hyaluronic acid, carboxymethylcellulose, or polyvinyl alcohol (PVA), encapsulate drugs that are released as the microneedles degrade [280]. Hollow microneedles, functioning like miniature hypodermic needles, facilitate liquid drug injection, making them particularly suitable for biologics and vaccines [271, 272]. Additionally, swelling and hydrogel-forming microneedles offer an alternative mechanism, where microneedles made from hydrophilic polymers absorb interstitial fluid upon application, swelling to form a gel-like matrix that allows controlled and sustained drug diffusion [281]. This technology is particularly advantageous for prolonged drug release applications and enhanced stability of hydrophilic drugs.

Fabrication techniques continue to evolve, optimizing scalability, precision, and cost-effectiveness. Micro-molding, one of the most widely used methods, involves casting biodegradable polymers into microneedle-shaped molds, ensuring high reproducibility and biocompatibility [271, 282]. Photolithography, adapted from semiconductor manufacturing, enables the production of ultra-precise microneedle arrays with uniform dimensions [282]. Laser micromachining, which uses high-intensity lasers to sculpt microneedles from metals, polymers, or silicon substrates, enhances structural stability [282–284]. Emerging fabrication methods such as electro-drawing, 3D printing, and centrifugal lithography are further expanding customization options, improving manufacturing efficiency and affordability, and enabling the development of multi-layered microneedle systems for complex drug delivery needs [271, 273, 285].

## The role of microneedle patches in modern drug and vaccine delivery systems: opportunities and challenges

In animal healthcare, microneedle patches represent a transformative alternative to conventional injection-based drug delivery, particularly for long-term therapies and livestock applications. Their minimally invasive nature reduces pain and stress, while controlled and sustained drug release improves treatment adherence, especially in cases of chronic illness. For instance, studies suggest that hormone replacement therapies for conditions such as adrenal insufficiency or hypothyroidism benefit from the consistent release profiles offered by microneedle platforms [286]. Research indicates that microneedle patches show significant promise for vaccination programs in veterinary species. By targeting the skin’s rich network of antigen-presenting cells, microneedles can boost immune responses comparable to traditional injections while reducing discomfort and handling stress, making them ideal for large-scale livestock immunizations [262] [287]. Their ability to deliver thermostable vaccines further addresses the challenge of cold-chain storage, which is often a limiting factor in remote or resource-limited environments [288, 289]. In addition, microneedles are being explored for pain management in animals, delivering sustained-release analgesics that improve comfort and welfare during postoperative recovery or chronic disease management [96, 290, 291]. Early studies in both small and large animal models demonstrate the feasibility of using dissolvable microneedles to administer NSAIDs and opioids in a controlled-release format, reducing the need for repeated injections and sedation.

Despite these advantages, several challenges must be addressed to support broader implementation in veterinary care. Transdermal drug delivery is strongly influenced by interspecies differences in skin thickness, lipid composition, follicle density, and hair coverage [246]. For example, porcine skin closely resembles human skin in thickness and permeability, whereas bovine and equine skin are substantially thicker and more fibrous, posing greater barriers to microneedle penetration [138, 246, 292, 293]. Companion animals exhibit high fur density and grooming behavior, further complicating patch adhesion and drug retention. These anatomical and behavioral differences necessitate species-specific microneedle design and application strategies. Species-specific differences in skin structure, including thickness, hydration, and hair/fur density, can impact microneedle penetration and drug diffusion, requiring tailored design strategies for each target species [1, 6, 250]. Accordingly, species-specific optimization of microneedle length and application pressure may be necessary to ensure effective skin insertion and consistent drug delivery, especially in animals with thick or highly elastic skin. In livestock and equine medicine in particular, variability in skin mechanics across anatomical sites further complicates device standardization. Accordingly, potential application sites for microneedle patches vary by species. In pigs, the ear and flank regions provide relatively accessible, low-mobility sites. In cattle, the neck and shoulder regions may be suitable, while in dogs and cats, regions with lower grooming access such as the dorsal cervical area are preferred. Anatomical site selection directly influences patch retention, drug absorption, and safety. While uniform penetration depth is a known concern in human applications, more veterinary-specific studies are needed to determine how skin elasticity, movement, and behavior influence patch performance across different animals. Similarly, drug stability remains a key limitation, especially for biologics, peptides, and protein-based therapies that are sensitive to heat, humidity, and oxidation [52, 294]. Innovative formulation strategies, including the use of stabilizing excipients, lyophilized drug cores, or encapsulated nanocarriers, are essential to preserve therapeutic efficacy during manufacturing, storage, and field application. Scalability and cost-effective production also remain barriers. Microneedle fabrication demands precise control over dimensions and mechanical strength, especially when devices are designed to be biodegradable or dissolvable. These features, while beneficial for safety and environmental sustainability, add complexity to mass production. Moreover, user compliance, such as ensuring proper application pressure and placement, can impact therapeutic outcomes, particularly in species with thick or highly mobile skin [295–298].

Lastly, regulatory approval for veterinary microneedle systems is in its early stages. Obtaining safety and efficacy data across multiple species requires significant investment in long-term trials. As the regulatory landscape evolves, continued research in polymer science, device engineering, and veterinary pharmacology will be essential in overcoming these adoption barriers.

Nonetheless, the future of microneedle drug delivery in veterinary medicine is highly promising. The development of biodegradable and dissolvable microneedle systems not only improves safety and environmental compatibility but also enables tailored release profiles for long-acting therapies. These innovations have the potential to redefine veterinary treatment paradigms, making drug delivery more precise, humane, and accessible, particularly in large-scale and remote animal health settings.

## Biodegradable microneedle patches: development, design, challenges, and opportunities

The development of biodegradable microneedle patches is centered on optimizing drug delivery while ensuring safety, effectiveness, and environmental sustainability [166]. Unlike conventional microneedles, which may require removal after use, biodegradable systems are designed to dissolve or degrade within the skin, eliminating the need for retrieval and minimizing medical waste. However, in practice, complete dissolution may not always be achieved within the intended application period, depending on factors such as microneedle composition, local skin hydration, animal movement, and anatomical site. Inconsistent application pressure or poor skin contact can also lead to partial insertion or incomplete dissolution, ultimately affecting drug delivery and biodegradability. Ongoing improvements in polymer formulation, application technique, and patch design are essential to enhance reproducibility and performance across species. In veterinary medicine, these features are especially valuable for improving treatment compliance and recovery, particularly in cases where repeated handling may compromise animal welfare.

Species-specific behaviors further influence the success of transdermal and microneedle-based drug delivery. Autolicking and heterolicking may remove applied formulations before adequate absorption, particularly in companion animals. Neophilia and neophobia can affect acceptance of novel devices such as patches, while coprophagia in certain species may influence exposure pathways [246, 292, 299–301]. These behaviors should be considered in the design of veterinary microneedle systems, particularly with respect to patch adhesion, selection of application sites, and the use of protective coverings.

A central aspect of microneedle design is material selection, as the polymer determines the needle's mechanical strength, biocompatibility, and degradation rate [302]. Synthetic polymers such as polylactic acid (PLA), polyglycolic acid (PGA), polylactic-co-glycolic acid (PLGA), and polyvinyl alcohol (PVA) are widely used due to their predictable degradation profiles and established safety in biomedical applications [303–306]. PLA and PLGA, in particular, have tunable degradation rates that allow for controlled drug release over time [306]. For example, PLGA's degradation rate can be adjusted by modifying the lactic-to-glycolic acid ratio, making it suitable for both short-term (e.g., post-surgical analgesia) and long-term (e.g., hormone therapy) veterinary applications. In contrast, natural biopolymers such as chitosan, gelatin, collagen, and alginate offer additional benefits, including enhanced biocompatibility, bioactivity, and wound-healing properties which may be particularly beneficial for animals prone to skin irritation or inflammation following patch application [303] Sugars like maltose, trehalose, and dextran are also used in microneedle formulations, especially for applications requiring rapid dissolution and immediate drug release. For instance, dextran is ideal for vaccine delivery in mass livestock immunization campaigns, where speed, dose consistency, and ease of administration are essential [307–309]. The choice of material must align with the intended application, whether it is for sustained drug release, rapid absorption, or structural stability during penetration.

Another key consideration in the development of biodegradable microneedles is drug incorporation. Drug incorporation presents additional challenges, particularly with biologic drugs such as vaccines, peptides, and proteins. The physicochemical properties of the drug such as solubility, stability, and molecular size play a determining role in its compatibility with microneedle systems [310, 311]. Hydrophilic drugs often suffer from instability and rapid degradation, necessitating encapsulation strategies like nanoparticles or liposomes to preserve their activity [312, 313]. For instance, in veterinary vaccine development, antigen-loaded nanoparticles integrated into dissolvable microneedles have shown improved thermostability and controlled antigen release, key for delivering consistent immune responses in remote or high-heat environments [314–316]. On the other hand, hydrophobic drugs, such as NSAIDs for pain management, often necessitate the inclusion of surfactants or emulsifying agents to facilitate uniform distribution within the microneedle matrix [317–319]. Some drugs require pre-treatment, such as lyophilization or chemical stabilization, before being integrated into microneedles to maintain their efficacy throughout storage and application [320, 321]. These factors influence both the release kinetics and overall therapeutic efficacy of the microneedle patch.

Fabrication techniques play a pivotal role in determining the mechanical performance and scalability of biodegradable microneedle patches [279, 322]. Mold-based microfabrication is one of the most commonly used approaches, where polymer solutions mixed with the active pharmaceutical ingredient are poured into microneedle molds, solidified, and then extracted to form the final patch. Enhanced versions like centrifugation-assisted micromolding improve drug distribution by pushing the solution deep into needle tips, which is especially important for achieving consistent dosing in veterinary applications [279, 323, 324].

Electrospinning, a method that forms microneedles from ultrafine fibers, allows high surface area and rapid drug release, suitable for topical or dermal veterinary therapies where localized, fast action is needed. Meanwhile, 3D printing enables customization of microneedle geometry, drug layers, and mechanical properties [14, 302, 325]. This is particularly advantageous for tailoring patch design to species-specific skin thickness or curvature, such as in cattle versus small companion animals. Techniques like droplet-born air blowing (DAB) and two-photon polymerization (TPP) offer microscale precision for creating complex drug-release profiles and hybrid structures but are still in early stages of translation to animal health [326, 327]. Finally, hydrogel-forming microneedles, which swell upon insertion to create sustained drug-release pathways, are being explored for extended pain management and hormone therapies in veterinary species. These systems maintain skin integrity while providing a depot-like mechanism that allows drugs to diffuse gradually from the swollen matrix [281, 328].

## Enhancing animal welfare and agricultural efficiency: advances, economic impact, and market development of microneedle patch technology

The integration of microneedle patches in veterinary medicine provides a less invasive and more efficient alternative to conventional drug administration, addressing critical concerns related to animal welfare, treatment compliance, and agricultural management [29]. Traditional injection-based drug delivery often induces stress, discomfort, and the risk of infection, particularly in livestock and companion animals requiring repeated treatments. Studies have shown that repeated handling and restraint for injections elevate cortisol levels and induce immunosuppressive effects, negatively impacting overall health and productivity [329]. By offering a pain-free, stress-reducing method, microneedle patches improve treatment adherence and contribute to better long-term health outcomes across various species.

The World Organization for Animal Health (WOAH) has emphasized the importance of innovative drug delivery systems to enhance compliance and support efforts to reduce antimicrobial resistance (AMR) in food-producing animals [330]. WOAH advocates for strategies that improve the responsible use of antimicrobials, minimize residues in animal products, and reduce the risk of resistance development. While microneedle patches are not explicitly mentioned in WOAH’s recommendations, their potential to enhance transdermal drug delivery, improve dosing accuracy, and reduce handling stress aligns with global efforts to develop alternative administration methods that support prudent antimicrobial use.

Beyond individual treatment benefits, microneedle technology offers significant advantages in large-scale agricultural settings. The ability to administer medications and vaccines without requiring specialized personnel reduces labor costs and improves efficiency in herd management [16, 21]. Additionally, eliminating needle waste and minimizing the risk of accidental needle-stick injuries enhance safety and sustainability in veterinary practice [1]. The economic benefits extend to livestock productivity and disease prevention, as healthier animals exhibit improved weight gain, higher milk yields, and better overall product quality [21, 331–334]. Effective disease prevention through microneedle-based vaccination reduces the need for expensive treatments and hospitalizations, supporting cost-effective farming operations. While the initial investment in microneedle patches may be higher than traditional injections, long-term savings in veterinary expenses, labor, and medication waste contribute to their cost-effectiveness.

The growing success of microneedle patches in human healthcare, particularly in chronic pain management, diabetes treatment, and non-invasive vaccine delivery, has accelerated interest in their veterinary applications [1, 335–338]. The cosmetic industry has also demonstrated their ability to enhance transdermal drug absorption while minimizing discomfort, further reinforcing their viability. This widespread adoption has driven further investment in biodegradable microneedle technology, with a focus on species-specific customization and long-acting drug formulations tailored to veterinary needs.

Despite their advantages, challenges remain in optimizing microneedle patch design for different species. Additionally, regulatory approval requires standardized safety and efficacy evaluations across multiple species to ensure consistency and compliance with veterinary drug administration guidelines. Addressing these concerns through continued research and regulatory advancements will be essential for expanding their use in both clinical and agricultural settings.

## Advancements and future directions in microneedle patch technology

Microneedle patch technology is rapidly evolving, offering innovative solutions for transdermal drug delivery in both veterinary and human medicine. These advancements focus on improving drug penetration, stability, and patient compliance while minimizing invasiveness. The continued refinement of microneedle designs and materials has the potential to innovate treatment strategies, particularly for conditions requiring sustained and controlled drug release.

One major area of research involves enhancing transdermal drug absorption through both chemical and physical strategies. The incorporation of chemical enhancers, such as alcohols, fatty acids, and surfactants, temporarily disrupts the skin barrier, increasing permeability and improving drug diffusion [310–312]. Similarly, encapsulation techniques using nanoparticles and liposomes protect active compounds from degradation while facilitating efficient absorption, ensuring more precise and prolonged drug release [313, 320]. Optimizing microneedle geometry adjusting needle shape, length, and density has also been shown to enhance penetration across varying skin types and species, reducing variability in drug delivery outcomes [279, 336].

In addition to these approaches, physical enhancement techniques such as sonophoresis (ultrasound) and iontophoresis (electrical stimulation) are being explored in combination with microneedle technology to further enhance transdermal absorption [175, 212, 339, 340]. The development of coated and hollow microneedles allows for precise dosing control, improving treatment accuracy, particularly for large molecules such as biologics, peptides, and nucleic acids [267, 268, 336]. These innovations are shaping the future of microneedle-based drug delivery, enabling their expansion into diverse therapeutic applications, including pain management.

Ongoing research has demonstrated that microneedle patches can facilitate the controlled transdermal administration of analgesics and nonsteroidal anti-inflammatory drugs (NSAIDs), offering a promising alternative to injection-based therapies [96, 341, 342]. Studies have shown that microneedle patches improve the bioavailability and therapeutic efficacy of NSAIDs such as meloxicam and ketoprofen, allowing for sustained pain relief with reduced dosing frequency [190, 343]. Our own research on microneedle-mediated NSAID delivery has further supported their potential for pain management in veterinary applications, highlighting their ability to enhance treatment adherence and mitigate stress in animals requiring long-term analgesic therapy [96].

Advancements in material science and formulation technology have played a key role in optimizing microneedle patches for pain management. Recent pharmacokinetic studies indicate that microneedle-administered NSAIDs maintain stable plasma concentrations over extended periods, reducing the need for frequent administration and improving compliance. For instance, a study on ibuprofen-loaded dissolving microneedle patches revealed a sustained drug release over 48 h, with a half-life significantly longer than that of traditional oral tablets [344]. Additionally, the integration of biodegradable and hydrogel-forming microneedles has addressed challenges related to drug release kinetics and patch removal, further enhancing their applicability in veterinary medicine [345]. These findings align with broader efforts to develop alternative pain management strategies that prioritize both efficacy and animal welfare.

As regulatory frameworks evolve, the clinical translation of microneedle patches for veterinary analgesia is becoming increasingly feasible. The growing body of preclinical and clinical data underscores their viability as a practical alternative to conventional NSAID administration, particularly in livestock and companion animals requiring sustained pain relief. The potential for smart microneedle systems, capable of releasing analgesics in response to inflammatory biomarkers, represents a significant step toward precision veterinary medicine.

The use of NSAIDs in managing post-procedural pain is particularly relevant in the context of routine animal husbandry practices, where effective analgesia remains a critical concern [343, 346–349]. In swine production, procedures such as castration and tail docking are routinely performed, often with limited pain management strategies due to logistical and economic constraints. As interest in improving animal welfare continues to grow, the development of alternative pain mitigation approaches such as transdermal meloxicam delivery via microneedle patches represents a promising solution [96]. Given the demonstrated efficacy of microneedle-based NSAID administration, further investigation into their role in addressing post-procedural pain in pigs is warranted.

These advancements underscore the growing potential of microneedle patches as a non-invasive, effective method for transdermal drug delivery, particularly for pain management in veterinary applications. As research continues to refine their design, optimize drug formulations, and address species-specific challenges, their role in improving therapeutic outcomes and animal welfare becomes increasingly evident. The next step lies in exploring their application in real-world scenarios, particularly in managing post-procedural pain in livestock, where effective analgesia remains a priority. Expanding the use of microneedle-mediated NSAID delivery could bridge the gap between innovation and practical implementation, offering a sustainable solution for improving both veterinary care and agricultural efficiency.

## Conclusions

Veterinary drug delivery continues to advance, improving treatment efficiency while prioritizing animal welfare and ease of administration. The selection of the appropriate administration route is crucial in achieving optimal therapeutic outcomes. While traditional oral and injectable formulations remain standard, emerging technologies such as transdermal patches and microneedle systems are transforming drug administration by reducing stress and enhancing compliance.

Microneedle patches, biodegradable polymers, and hydrogels provide controlled, sustained drug release, minimizing the need for frequent dosing. These innovations improve treatment adherence, particularly in chronic conditions and large-scale livestock applications. However, the success of these technologies depends on proper education for veterinary professionals and caregivers, as well as regulatory approval to ensure safety and efficacy across species.

The future of veterinary drug delivery lies in continuous research and the integration of advanced biomaterials and targeted therapies. By bridging technological advancements with practical applications, veterinary medicine is moving toward more effective, stress-free, and sustainable treatment solutions. These innovations will enhance overall veterinary care, benefiting both companion animals and livestock while supporting public health initiatives.

## Data Availability

N/A.
